# A Novel Antenna for Partial Discharge Measurements in GIS Based on Magnetic Field Detection

**DOI:** 10.3390/s19040858

**Published:** 2019-02-19

**Authors:** Armando Rodrigo-Mor, Fabio Andrés Muñoz, Luis Carlos Castro-Heredia

**Affiliations:** Electrical Sustainable Energy, Delft University of Technology, 2600 GA Delft, The Netherlands; A.RodrigoMor@tudelft.nl (A.R.-M.); L.C.CastroHeredia@tudelft.nl (L.C.C.-H.)

**Keywords:** partial discharge (PD), GIS, HVAC, magnetic field antenna, sensitivity check, pick-up coil

## Abstract

This paper presents a new concept for partial discharge measurements in gas insulated systems (GIS). The proposed technique uses a novel GIS magnetic antenna that measures the magnetic field produced by partial discharges (PD) propagating in GIS. The foundations of the measurement technique and the magnetic antenna design are presented together with laboratory measurements. The magnetic antenna performance and the sensitivity of the acquisition system are studied. The bandwidth of the measurement system is in the high frequency and very high frequency (HF–VHF) range. Laboratory experiments demonstrate the suitability of the novel magnetic antenna-based measuring system for PD in GIS for corona, surface discharges, and free moving particles in SF_6_.

## 1. Introduction

Nowadays, partial discharge measurement plays an important role in the monitoring and diagnostic of high voltage gas insulated systems (GIS) due to its high capabilities to detect in-service failures related to defects in the insulation system [1].

Partial discharges in a GIS produce electromagnetic waves of different modes that travel along the coaxial structure formed by the enclosure and the main conductor. The generated electromagnetic wave includes transverse electric (TE) and transverse magnetic (TM) modes in addition to the transverse electric and magnetic (TEM) mode. At low frequency (up to hundreds of MHz) the TEM mode is predominant, whereas, at high frequencies, the TE11 is the most significant mode with a cut off frequency in the range of hundreds of MHz [2].

Since its introduction, back in the 80 s, the ultra-high frequency (UHF) method (300 MHz to 3 GHz) has been the most extensively used for partial discharge measurements in GIS, because it is more resilient to noise and easier to handle in comparison with the IEC conventional method [1]. The UHF system is based on the measuring of the ultra-high frequency components of the electric field caused by a partial discharge. Therefore, a UHF antenna captures the signals mostly in the range in which the TE11 is the predominant mode of propagation. Nevertheless, in [3,4,5,6,7,8] it was found that the electromagnetic waves in a GIS structure suffer from attenuation and distortion mainly in the higher order modes (TE11). For instance, in a T branch structure, the TE11 component is heavily attenuated and distorted due to the multiple reflections [4], whereas the TEM mode travels according to the transmission line theory, and as a result, the current is distributed based on the characteristic impedance of the T branch. As a consequence, the sensitivity of a UHF measuring system substantially decays with the distance between the partial discharges (PD) source and the sensor [1].

On the other hand, a PD pulse creates an electric current that travels along the inner conductor (main conductor) and the outer conductor (enclosure). This PD current flows mainly on the surface of the conductors (main conductor and enclosure) due to its high-frequency spectrum and the skin effect. As the electromagnetic wave, this current also suffers more attenuation and distortion at high frequencies than at low frequencies. 

A novel PD measuring system for GIS, based on high-frequency transformers (HFCT), has been recently developed by the authors [9]. This system measures the PD currents flowing along the GIS enclosure at the spacer locations. However, for the time being, the HFCT-based system is only suitable for external spacers, requiring design modifications of the actual internal spacers if it is to be used in current GIS.

The detection of currents in the enclosure paved the way for a new PD measuring system for GIS based on a novel magnetic field antenna. This design has the advantage that the magnetic antenna can be installed in conventional dielectric windows in which traditional UHF antennas are installed. 

## 2. Magnetic Field Detection of Currents in Openings in Coaxial Structures

In a GIS system, partial discharges radiate electromagnetic waves in a frequency spectrum ranging from DC to GHz. These electromagnetic waves induce surface currents in the main conductor and in the external enclosure. The PD surface current flows along the compartments, the electrical paths in the spacers, the L branches, T joints, circuits breakers or any other electrical path in the GIS while suffering from attenuation, reflections, and dispersion. 

**Figure 1:**
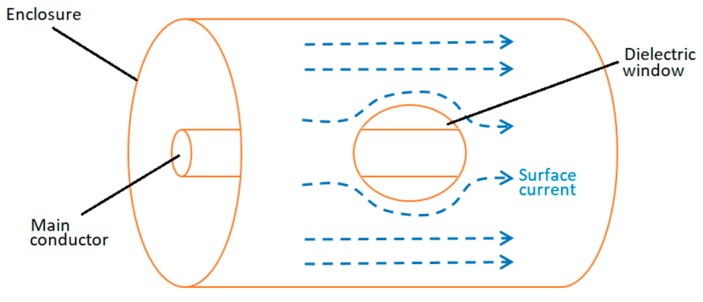
Current deviation around the window.

In [9], it has been demonstrated that in a GIS compartment, such as the one shown in Figure 1, the surface current is homogeneously distributed in the circumference formed by the cylindrical enclosure. However, in an enclosure with dielectric windows that are usually used to allocate VHF/UHF antennas, the PD current has to travel around the window. 

In a coaxial structure, a homogenously distributed surface current does not create a resultant magnetic field outside the external conductor (enclosure). However, the deviation of the current around the dielectric window produces a magnetic field perpendicular to the contour of the window. Additionally, considering the transient behavior of the PD current, this magnetic field is time-varying. 

**Figure 2:**
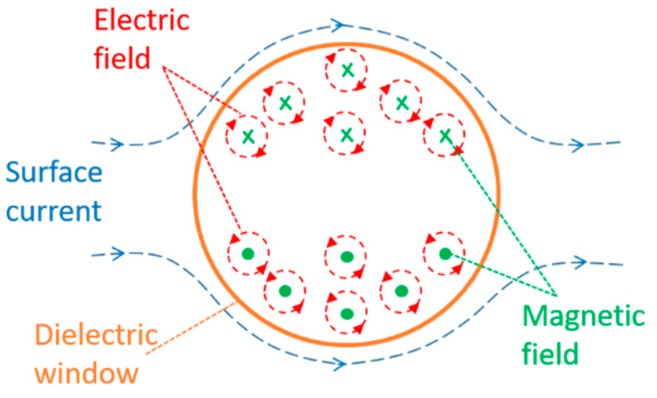
Induced electric field in the dielectric window.

Based on Faraday’s law, Equation (1), the time-varying magnetic field in the dielectric window creates an induced electric field in the surface. This induced electric field is illustrated in Figure 2.

(1) $$\nabla x{\vec{E}}_{ind}=-\frac{\partial \vec{B}}{\partial t}$$

Considering the non-conservative trait of the induced electric field, it is possible to produce an electromotive force (EMF), Equation (2), in a wire placed in a closed curve in the region that forms the dielectric window.

(2) $$\xi =\oint {\vec{E}}_{ind}\;d\vec{l}$$

Therefore, the surface current generated by a PD pulse can produce an induced voltage in a hypothetical loop antenna placed in the dielectric window. Nevertheless, it is necessary to study in detail the physical phenomenon to know, for instance, the real distribution of the current, the magnetic field magnitude and direction, and the magnitude and shape of the induced voltage. For this purpose, a Comsol simulation of a PD pulse traveling in a GIS model is presented in the following section.

### Fundamentals

**Figure 3:**
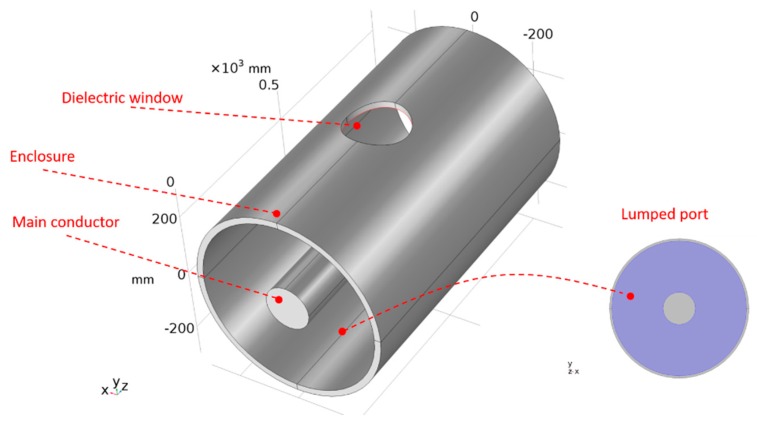
Simulation model geometry.

In this section, the electromagnetic wave propagation in a GIS was tested using a finite element model based on a simple GIS compartment without spacers, radio-frequency physics, and a time domain study. Figure 3 shows the simulation model geometry. The model was made with a cylindrical main conductor, a cylindrical enclosure and a dielectric window in the enclosure. The dielectric window was located in the middle of the length of the enclosure to allow symmetrical propagation of the electromagnetic wave before it reaches the window. The dimensions of the model are given in Table 1. 

**Figure 4:**
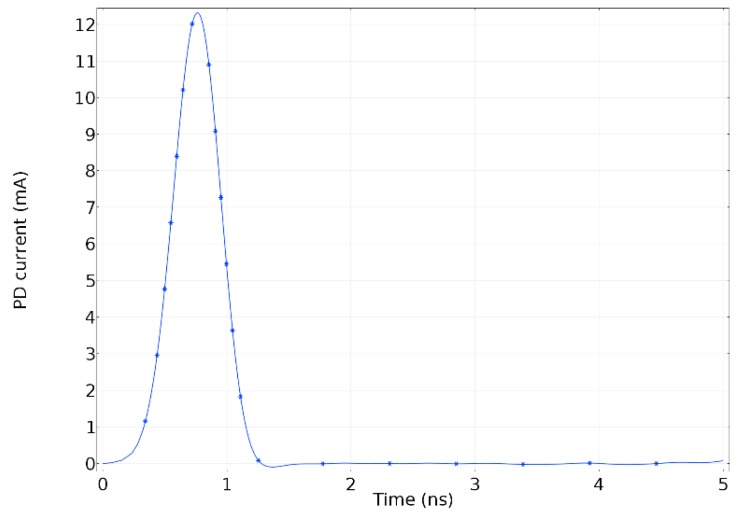
Partial discharge (PD) pulse injected.

The excitation of the electromagnetic wave was done through a coaxial lumped port assigned to the left end of the model presented in Figure 3. The lumped port was assigned to the dielectric boundary shaped by the main conductor and the enclosure. This lumped port was set-up with the PD pulse presented in Figure 4 and perfectly matched based on the characteristic impedance of the compartment. The other end, the right-hand side, was set with a coaxial lumped port perfectly matched as well to the characteristic impedance of the coaxial structure. The wave excitation of this port was set in the condition off. 

To speed up the numerical calculations, the conductor elements (the enclosure and the main conductor) were modelled as perfect electric conductors. 

Regarding the simulation mesh, the mesh statistics, and size are presented in Table 2. 

To assure the convergence and the exactitude of the numerical results, the time-dependent study was set with a relative tolerance of 1 × 10^−6^; the time-dependent solver was set up with a Pardiso linear solver, the time stepping with the generalized-alpha method, and the time range was adjusted between 0 ns and 5 ns in steps of 0.01 ns.

**Figure 5:**
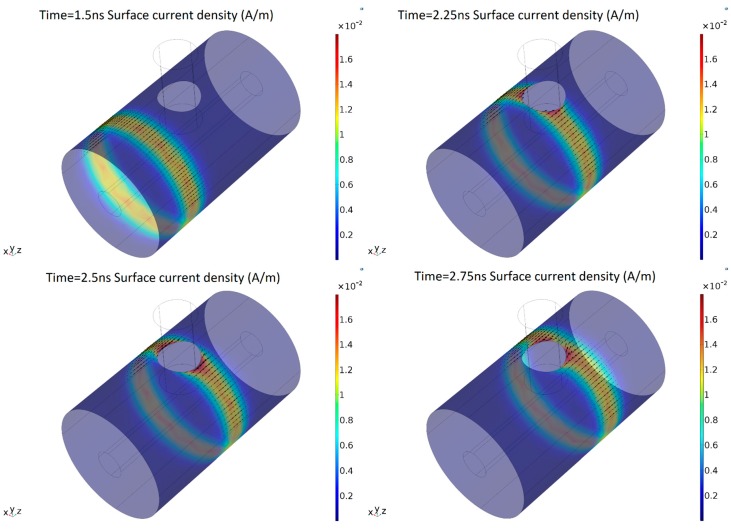
Surface current temporal evolution.

The results of the simulation have shown that the PD pulse induces a surface current in the main conductor and in the enclosure, which travels along the whole compartment. The evolution of the surface current in the enclosure is shown in Figure 5. It is possible to see that at 1.5 ns before the pulse reaches the window, the current is homogeneously distributed around the external cylinder. Later, when the current arrives at the dielectric window at t = 2.25 ns, it suffers a deviation around the window. It is also worth noting that there is a higher current density at both sides of the window in comparison with the rest of the enclosure, where the surface current density is still relatively homogeneous. 

**Figure 6:**
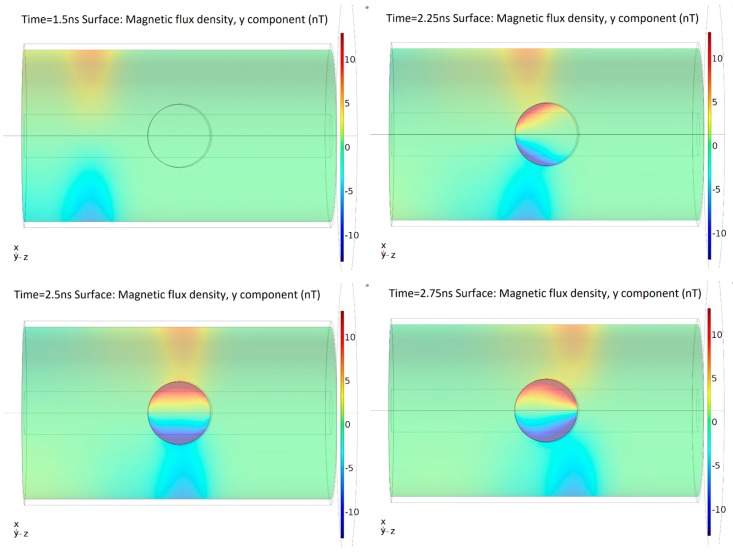
Magnetic flux density perpendicular to the dielectric window at different times.

As expected, the surface current creates a magnetic field normal to the surface shaped by the dielectric window. Figure 6 shows the time evolution of the magnetic flux density in the *y*-axis, which is the direction perpendicular to the dielectric window.

In the dielectric window, for each instant of time, the spatial distribution of the magnetic field has an odd-symmetry with respect to the *y*-axis, as is remarked in Figure 6. This means that both half-windows have the same magnetic field magnitude and distribution but in the opposite direction. Additionally, the magnetic field density is higher near the dielectric window border. 

**Figure 7:**
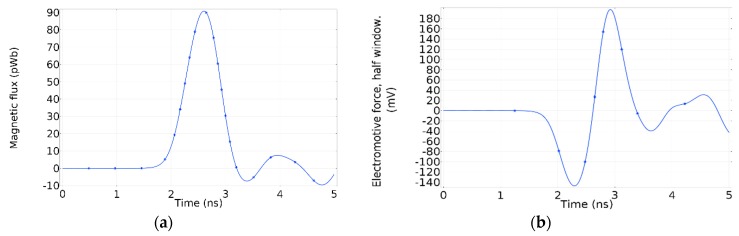
(**a**) Magnetic flux through half-window; (**b**) electromotive force induced in half-window.

Due to the magnetic field distribution in the window, the total magnetic flux through the whole surface is equal to zero, which means there is no resultant flux that could generate an electromotive force in the whole window. However, due to the odd-symmetry, in a half-window, there is a resultant magnetic flux. Therefore, each half-window has an electromotive force with the opposite polarity. In Figure 7a, the resultant magnetic flux crossing half-window is shown. The electromotive force induced in half-window by the magnetic flux is shown in Figure 7b.

In conclusion, the finite element (FEM) simulation has proved that the PD current can generate an induced voltage in the dielectric window, where the traditional VHF/UHF antenna for partial discharges detection is placed. This fact provides a way to develop a new measuring system of partial discharges based on the induced voltage measurement through a magnetic loop antenna placed in half-window.

## 3. The Magnetic Antenna

The magnetic antenna consists of two shielded pickup coils connected to their own high input impedance operational amplifier. 

**Figure 8:**
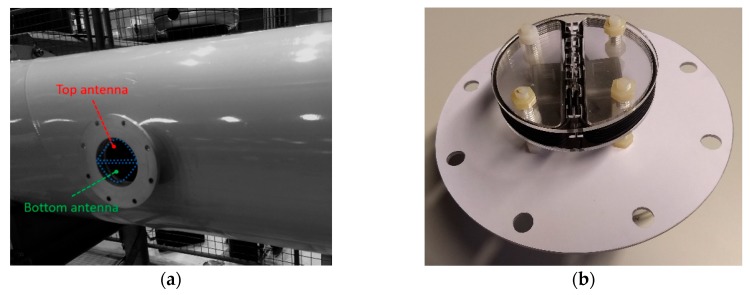

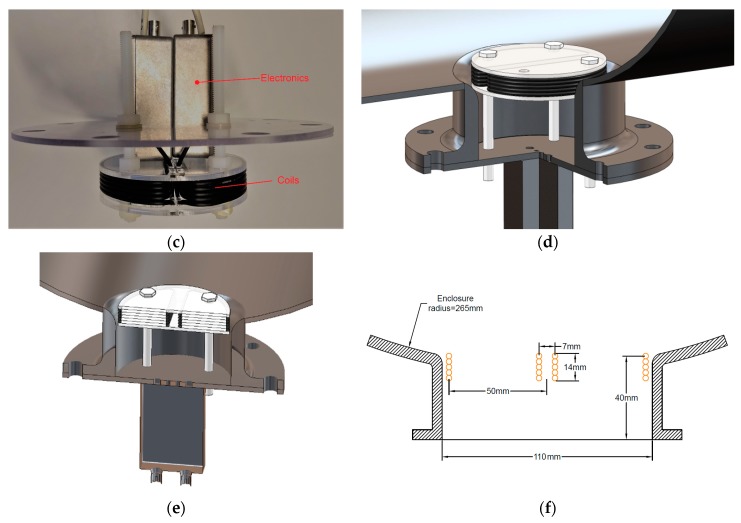
(**a**) Gas insulated system (GIS) enclosure and dielectric window opening and pick-up coils direction in dielectric window (**b**) Magnetic antenna; (**c**) magnetic antenna detail; (**d**) perspective view of magnetic antenna installation; (**e**) cutaway view of magnetic antenna installed in the GIS compartment; (**f**) axial cross section of the dielectric window and the antenna coils with dimensions.

Each pickup coil is a five turns loop coil wounded in a half-circle shape, see Figure 8b. The coils are installed in a symmetrical arrangement, with the straight lines of the half-circle shapes running parallel to the GIS axis, as is shown in Figure 8a,e. This configuration allows to pick up the magnetic flux as depicted in Figure 6. Moreover, the coils are made using coaxial cable to provide electric field screening. 

In this case, RG173 type coaxial cable has been used to make the coils. The inductance of each coil is 2.5 μH, while the capacitance accounts for 126 pF. The radius of the coils is 50 mm. In the dielectric window, the coils were fixed as close as possible to the inner diameter of the enclosure to collect the magnetic field produced by the PD. Figure 8f shows the dimensions and position of the pick-up coils in the dielectric window. The coax cable screen is grounded at one cable end, and unconnected in the other. In this configuration, the screen of the coax cable electrically shields the inner loop, formed by the coax cable inner conductor, thus, preventing the electric field from coupling with the inner coil. 

**Figure 9:**
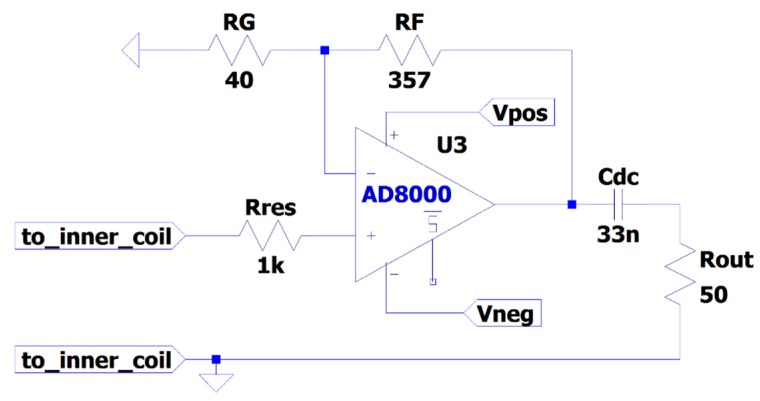
Magnetic antenna RF amplifier schematic.

The inner coil, formed by the inner conductor of the coax cable, is grounded at one end, while the other end is connected to a high impedance operational amplifier. The op amp is connected in a non-inverting amplifier configuration with a 10 times gain. A 1 kΩ series resistor is connected to the non-inverting input to prevent oscillations due to resonance effects of the combined loop inductance and the internal input capacitance of the op amp. Moreover, a dc decoupling capacitor is placed at the op amp output to block the offset voltage at the output. Figure 9 shows the radio frequency (RF) amplifier scheme of the magnetic antenna with component values. Figure 8c shows the boxes where the amplifiers where installed. 

## 4. Laboratory Experiments

The experiments were conducted on a 380 kV SF_6_ gas insulated system at the High Voltage Laboratory of TU Delft. The GIS has multiple spacers, a T-joint connection, an earth switch, a switchgear, a bushing, an L-branch structure, a disconnector switch and eight dielectric windows for UHF PD testing with a diameter of 110 mm. 

**Figure 10:**
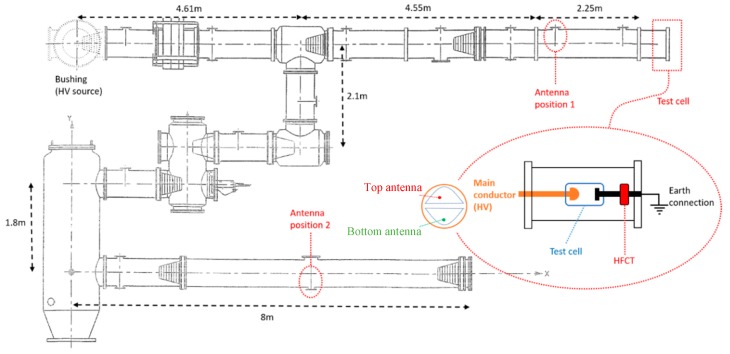
GIS layout with magnetic antenna locations.

A set of three test cells were deployed to produce corona, surface, and free moving particle discharges. Each test cell was built under SF_6_ pressure and was installed in the GIS compartment as is shown in Figure 10. In the rod holding test cell, a high frequency current transformer (HFCT) was installed to measure the PD current at its source, which allows for charge estimation according to [10]. For the corona and surface test cells, a 25.5 dB amplifier was connected at the output of the HFCT to improve the signal to noise ratio (SNR). The HFCT has a gain of 9.1 mV/mA and bandwidth from 62 kHz to 136 MHz. Further information about the HFCT can be found in [11]. Test voltages and SF6 pressures for each test are summarized in Table 3.

In the experiments, the UHF antennas located at position 1 and 2 in Figure 10 were dismounted and the magnetic antennas introduced in this paper were installed instead. At each position, a top antenna and a bottom antenna were symmetrically fit into the dielectric window with the orientation indicated in Figure 8a and Figure 10.

A Tektronix MSO58 oscilloscope set at 1.5625 GSa/s was deployed to record the PD signals. The acquisition of the signals coming from the sensors was made using the Fast Frame Acquisition Mode because it allows capturing multiple PD pulses at the sampling rate mentioned before. Each PD pulse was recorded individually in a frame with a record length of 4 µs.

Due to the geometries of every test cell, different test voltages and SF_6_ pressures were needed for each test as can be seen in Table 3.

### 4.1. Measurements with Artificial Defects

**Figure 11:**
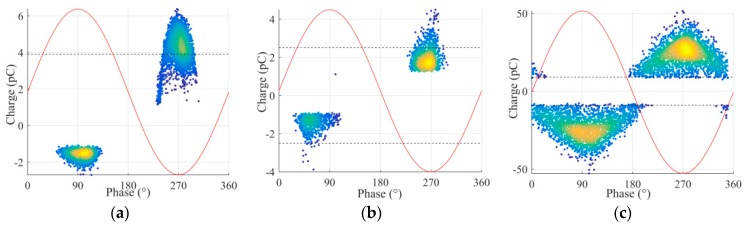
PRPD patterns, (**a**) corona, (**b**) surface, (**c**) free moving particle.

A collection of measurement results is reported in this section to verify the performance of the magnetic antenna. The laboratory experiments consisted of using the test cells to generate PD signals at one end of the GIS and record the signal from the HFCT and from the antenna simultaneously. The measurements were conducted first with the antenna at position 1 (test 1) and later at position 2 (test 2) always utilizing the HFCT signals as the trigger source of the oscilloscope. The phase resolved partial discharge (PRPD) patterns obtained from the HFCT in test 1 are shown in Figure 11.

Then, by using the software *PDflex* [12] the signals from the antenna were compared to the signals corresponding to every point in the PRPD pattern to verify if the antenna signals corresponded to actual PD pulses or disturbances/noise. A signal from the antenna was considered a PD signal when it met these three criteria: Its peak occurred around the same time of the peak of the HFCT signal, its peak amplitude was roughly two times the peak of the background noise (from the starting of the recording to the time of its peak), and the cumulative instantaneous power of the measured signals indicates a clear pulse. 

The cumulative instantaneous power is defined in Equation (3).

(3) $$P\left[t\right]=f_{s}\times \frac{1}{N}\overset{N-1}{\underset{n=0}{\sum }}s^{2}\left[n\right]\;V^{2}/s$$

where *s*[*n*] is the measured PD signal in the antennas, *f_s_* is the sampling frequency of the signals (1.5625 GS/s), and *N* is the number of samples (4 µs × 1.5625 GSa/s = 6250 Sa). 

**Figure 12:**
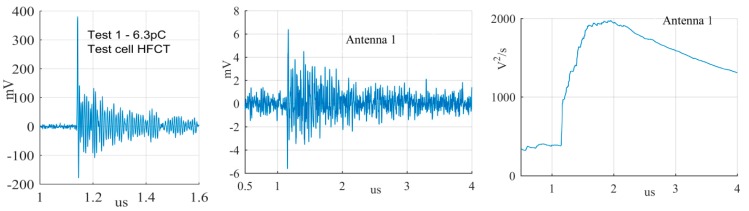

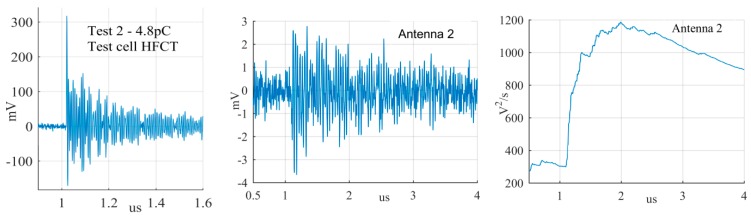
Largest corona discharge from the antenna at position 1 and 2.

**Figure 13:**
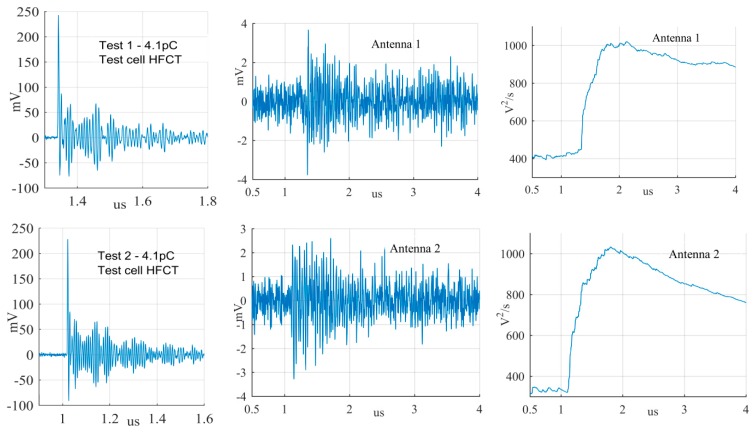
Largest surface discharge from the antenna at position 1 and 2.

**Figure 14:**
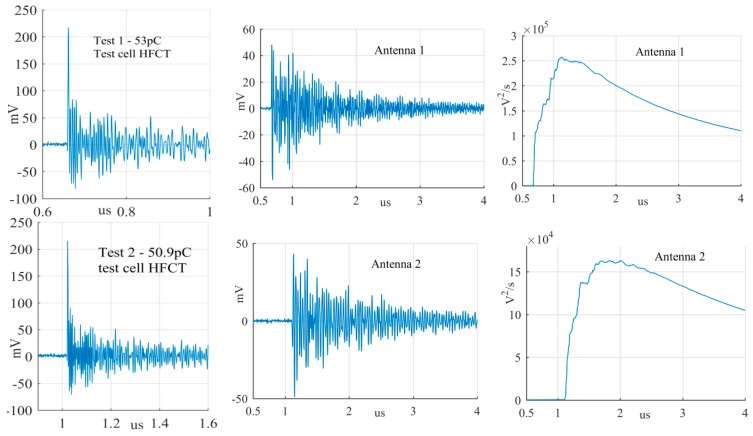
Largest free moving particle discharge from the antenna at position 1 and 2.

**Figure 15:**
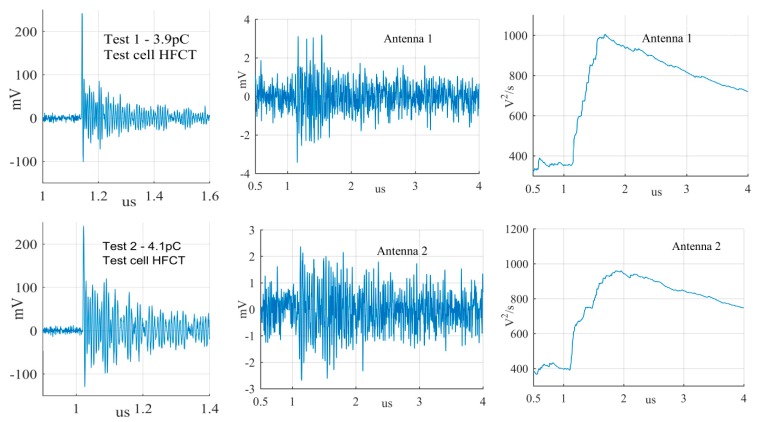
Smallest corona discharge from the antenna at position 1 and 2.

**Figure 16:**
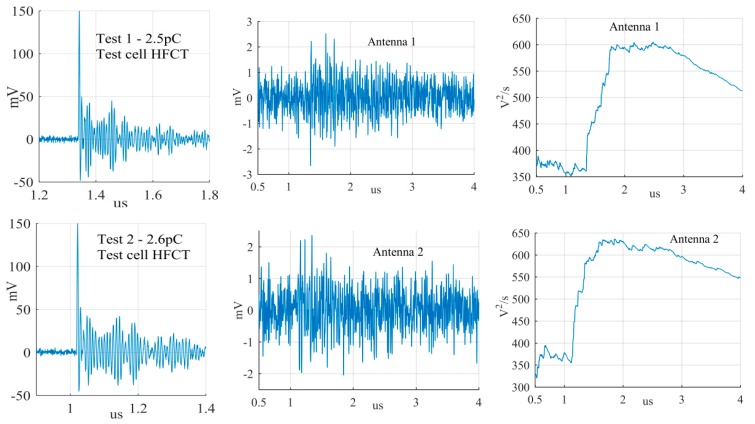
Smallest surface discharge from the antenna at position 1 and 2.

**Figure 17:**
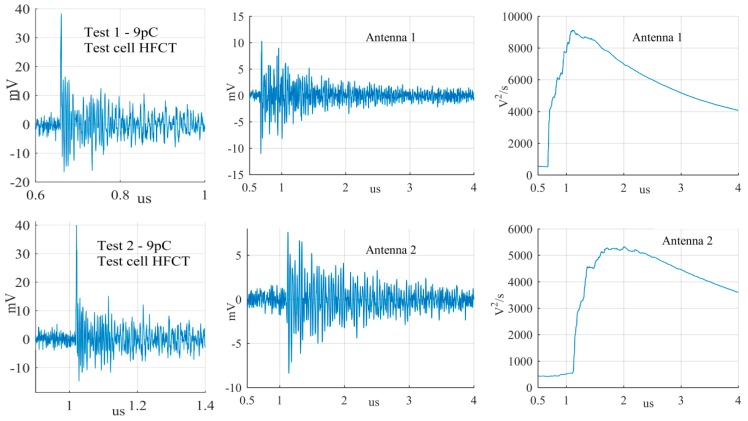
Smallest free moving particle discharge from the antenna at position 1 and 2.

The largest PD signals from the patterns in Figure 11 are shown in Figure 12, Figure 13 and Figure 14, whereas the smallest signals meeting the above criteria are depicted in Figure 15, Figure 16 and Figure 17. The figures show the PD pulse recorded with the HFCT at the test cell, the antenna signals and the cumulative instantaneous power *P*[*t*] of the antenna signals.

Table 4 summarizes the criteria applied to the largest and smallest signals for both antenna positions.

The power of the signals depicted in Figure 12, Figure 13, Figure 14, Figure 15, Figure 16 and Figure 17 show two different zones: a flat zone at the beginning of the signal, followed by a significant rising of the power. The flat zone corresponds to the background noise (P_n_) that shows has constant power. The notable increase of the power after the flat zone indicates the presence of the PD signal; the end of the flat zone is defined as the background noise just before the rising of the power (P_n_), and the maximum power of the measured signal is termed as P_p_. In Table 4, the ratios between the P_p_ and the P_n_ are shown. The power ratio is higher in antenna 1 than antenna 2 (except for the smallest surface), which is explained by the fact that antenna 1 is closer to the source than antenna 2. 

The criteria were applied to all the signals, and then a minimum PD magnitude was determined for each defect. In the PRPD patterns of Figure 11, thresholds lines were added to indicate the minimum PD amplitude that led to signals in the antennas meeting the criteria for being identified as PD signals. 

### 4.2. Magnetic Field Symmetry

The FEM simulation has shown that each half-window has the same electromotive force, but with the opposite polarity, which means that the top antenna and the bottom antenna may have the same voltage magnitude but with opposite polarity. 

**Figure 18:**
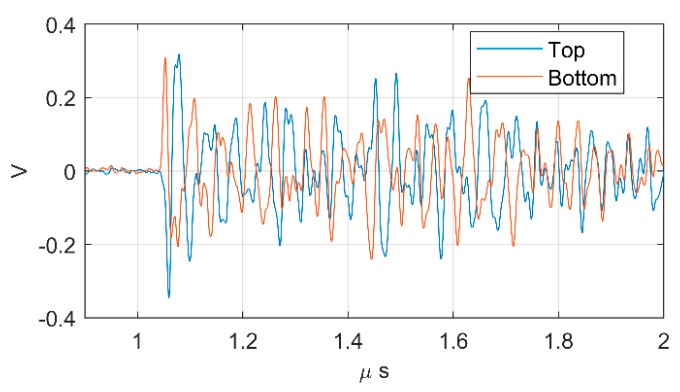
Comparison between the top antenna and the bottom antenna of a measured PD.

To check this simulation result, the signals coming from the top and the bottom antenna were compared to a real PD. An example of this comparison is shown in Figure 18, where it is noticed that the first peaks have almost the same magnitude but opposite polarity. Differences between both antennas could be due to the limitations of the electric field screening. 

**Figure 19:**
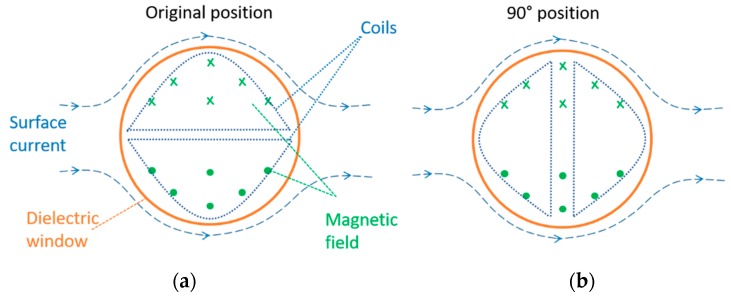
(**a**) Antenna in normal position. (**b**) Rotated antenna 90 degrees.

To test the magnetic performance of the antenna an additional measurement was performed at position 2 using the corona test cell. In this measurement, the antenna was rotated 90 degrees clockwise in the dielectric window, as is illustrated in Figure 19b. With this configuration, the induced voltage would be zero because the total magnetic flux is zero. 

**Figure 20:**
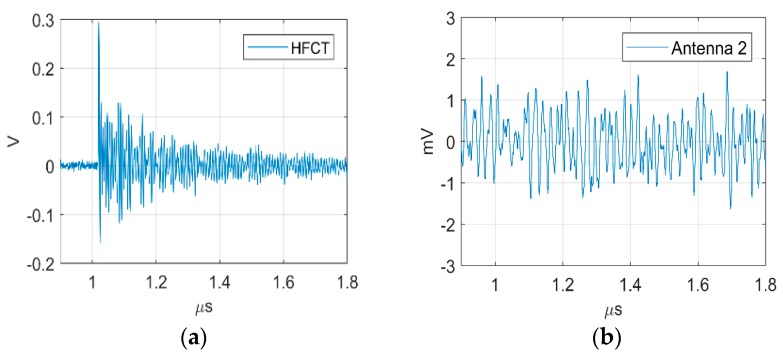
(**a**) Largest corona discharge HFCT; (**b**) Largest corona in the antenna at position 2.

Figure 20 shows the largest PD signal recorded at the HFCT and its corresponding signal at the antenna. Although the HFCT is measuring the largest PD signal, the antenna is only recording background noise, which means that there is no induced voltage at the antenna. This result confirms the fact that the top antenna and the bottom antenna are measuring—mainly—the magnetic field at the dielectric window. 

### 4.3. Magnetic Antenna Frequency Response

**Figure 21:**
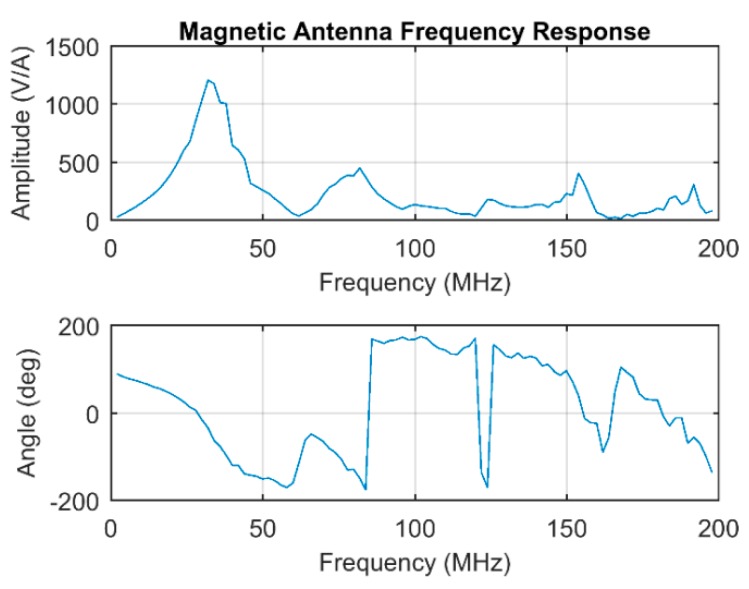
Magnetic antenna frequency response.

To characterize the frequency response of the magnetic antenna, a single loop with the same shape of the pick-up coil has been placed 3 mm above the top of the antenna. A signal generator injecting a current pulse with a frequency content in excess of 200 MHz has been connected to the single loop to induce a magnetic field in the five turns pick-up coil. The frequency response of the antenna has been derived from the Fast Fourier Transform (FFT) of the injected current pulse and the FFT of the measured voltage signal with the antenna. The bode magnitude and phase plots are depicted in Figure 21.

The Bode magnitude plot shows the typical response of an inductive measuring system with parasitic capacitances which creates multiple resonances. The main antenna resonance is around 32 MHz, and it is mainly associated with the pick-up coil inductance and the coaxial cable capacitance. 

### 4.4. PD Pulse Spectrum

**Figure 22:**
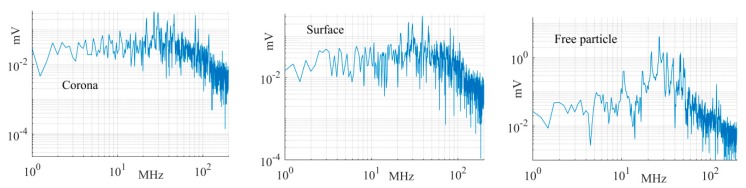
Frequency spectra of the largest signals measured by the antenna at position 1.

Figure 22 depicts the frequency spectrum of the largest PD signals measured by the magnetic antenna at position 1 for the corona, surface, and free moving particle test cells. It is clearly shown that the spectrum is below 100 MHz in all cases. It is worth mentioning that the frequency spectrum is associated with each PD pulse and its multiple reflections in the GIS. 

## 5. Discussion

The magnetic antenna design has shown its capability in measuring the magnetic field produced by the partial discharges. 

However, there are still remaining issues to be addressed, such as the influence of the dielectric window size, the number of turns, and the resilience to the noise. 

**Figure 23:**
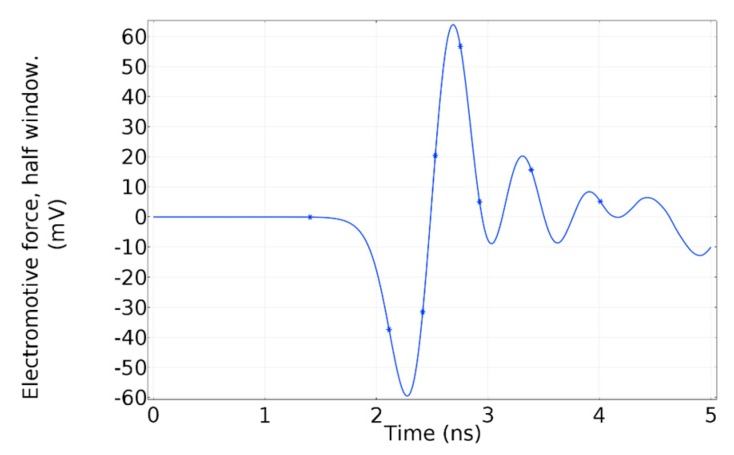
Electromotive force induced in half-window of a dielectric window with a radius of 50 mm.

As has been mentioned, the surface current in the enclosure suffers a deviation around the dielectric window, which creates the time-varying magnetic field on the surface formed by the dielectric window. Moreover, this current deviation is proportional to the dielectric window size because the remaining current preserves its homogeneous distribution around the enclosure. Therefore, it is conceivable to assume that the dielectric window size has a relevant influence on the voltage induced in the magnetic antenna. To have a better understanding of the physical phenomenon behind it, a Comsol simulation was done with the same characteristics of the previous one, but having a dielectric window with a radius of 50 mm instead of 100 mm. The voltage induced in half-window is shown in Figure 23. 

A quick glance at the electromotive force induced in the dielectric window with 50 mm radius, reveals that the amplitude has substantially decreased in comparison to the induced voltage in a dielectric window with a radius of 100 mm. In Figure 23, the first peak value is around −60 mV, whereas in Figure 7b it is −140 mV. Nevertheless, further experiments need to be done to confirm these previous results.

It is worth mentioning that the antenna deployed had five turns, a planar geometry, and concentric coils; however, it is possible to have different configurations that may allow the antenna to pick up the magnetic field with higher sensitivity. Consequently, further study on the antenna design has to be conducted to check for improvements.

Finally, it is necessary to study the effect of the noise in the magnetic antenna in situations with high background noise.

## 6. Conclusions

This work contributes an alternative approach to measure PD signals in GIS based on magnetic field detection. This technique seizes the magnetic field created in the dielectric windows of the GIS compartments to induce an EMF in a pick-up coil or magnetic antenna. 

The antenna and the measuring arrangement are simple, making use of the existing openings of the GIS. Therefore, in comparison with [9], no modifications of the GIS are required. Experimental results showed that the ratio between the PD signal and the noise level at location 2 (~20 m from the test cell) was of the same order of magnitude as that at location 1 (~3 m from the test cell).

The antenna performance has been evaluated by direct comparison with the induced PD currents in the GIS produced by corona, surface, and free moving particle test cells. Discharges of a few pico Coulombs have been successfully measured, which demonstrates the suitability of the measuring system per se.

Compared to conventional VHF/UHF measurements, this antenna works in the HF–VHF range meaning that implementation at system level would require lower specifications from the acquisition units. 

Nevertheless, such a low-frequency range also may make the detection vulnerable to noise and disturbances. Yet, clustering or post-processing techniques can be applied to overcome these issues. 

## Figures and Tables

**Table 1:** Model dimensions.

Component	Radius	Length
Enclosure	300 mm	1000 mm
Main conductor	70 mm	1000 mm
Dielectric window	100 mm	---

**Table 2:** Mesh statistics and size.

Mesh Statistics		Mesh Size	
Minimum element quality	0.2268	Maximum element size	82.5 mm
Average element quality	0.6841	Minimum element size	6 mm
Tetrahedron	54783	Curvature factor	0.4
Triangle	5432	Resolution of narrow regions	0.7 mm
Edge element	499	Maximum element growth rate	1.4
Vertex element	36	Predefined size	Finer

**Table 3:** Test parameters.

		Corona	Surface	Free Moving Particle
AC test voltage		15 kV	20 kV	12 kV
SF6		3 Bar	1.2 Bar	2 Bar
Test cell	sensor	gain 9.1 mV/mA, BW 62 kHz–136 MHz		
	amplifier	25.5 dB, 23 kHz–1.14 GHz		--
Antenna 1: position at 2.3 m from test cell (**test 1**) Antenna 2: position at 20 m from test cell (**test 2**) Trigger of acquisition: HFCT at the test cell				

**Table 4:** Power and amplitudes ratios.

	Ratio P_p_/P_n_		Ratio Peak Amplitude/Peak Background Noise	
	Largest Signals	Smallest Signals	Largest Signals	Smallest Signals
Corona				
antenna 1	5.1	2.8	4.6	1.6
antenna 2	3.8	2.6	2	2.6
Surface				
antenna 1	2.3	1.7	2.6	2.1
antenna 2	3.2	1.8	2.1	2
Free moving particle				
antenna 1	662.4	17.8	84.6	9.4
antenna 2	327.4	9.7	31	4.7
